# Physical Properties of Mold Flux and Mineralogical Characteristics of Flux Film for Low-alloy Peritectic Steel Continuous Casting

**DOI:** 10.3390/ma18184298

**Published:** 2025-09-13

**Authors:** Di Zhang, Xiuli Han, Lei Liu, Jingjing Guo, Yue Yang, Lei Wu

**Affiliations:** 1 College of Mining Engineering, North China University of Science and Technology, Tangshan 063210, China; zhangdi@ncst.edu.cn (D.Z.); liulei9200@ncst.edu.cn (L.L.);; 2 Collaborative Innovation Center of Green Development and Ecological Restoration of Mineral Resources, Tangshan 063210, China; 3 College of Metallurgy and Energy, North China University of Science and Technology, Tangshan 063210, China

**Keywords:** mold flux, melting properties, crystallization behavior, mineralogical structure, heat transfer mechanism, low-alloy peritectic steel

## Abstract

To meet the demanding requirements of continuous casting for low-alloy peritectic steel, this study aimed to design high-performance mold fluxes with optimized properties. The melting properties, crystallization behavior, mineralogical characteristics, and heat transfer mechanism of the industrial mold fluxes and flux films were investigated by melting tester, viscometer, in situ thermal analyzer, thermal conductivity meter, polarizing microscope, X-ray diffraction, and thermodynamic software. The results demonstrate that mold fluxes suitable for low-alloy peritectic steel possess a narrow melting temperature range, low melting point (<1200 °C), and low viscosity (<0.1 Pa·s) to ensure adequate fluidity and lubrication. A key characteristic of the mold fluxes is strong crystallization ability, reflected by a high critical crystallization cooling rate (>50 °C/s) and high initial crystallization temperature (>1350 °C), facilitating the rapid formation of a stable crystalline layer and uniform heat transfer. The flux films with outstanding characteristics have a multilayered structure and high crystallization ratio (60–80 vol%), predominantly comprising a high fraction of coarsened cuspidine crystals. Further analysis of the heat transfer mechanism reveals that the highly crystalline and coarse-grained microstructure promotes the formation of micropores and crystal boundaries in flux films, which substantially increase thermal resistance, leading to low thermal conductivity (0.47–0.67 W/m·K) and effective control of heat transfer rate. It is concluded that enhancing crystallization performance through optimizing flux composition (boosting Na_2_O content and basicity) to promote cuspidine formation and tailor crystallinity, is the crucial route for acquiring the desired mineralogical structure of flux films and enabling efficient continuous casting of low-alloy peritectic steel.

## 1. Introduction

Mold flux, as a key functional material in the continuous casting process, is crucial for ensuring smooth operation and high-quality slabs [1,2,3]. Currently, significant issues persist in the selection and application of mold fluxes at steel plants, hindering their ability to adequately meet the requirements for casting exceptional steel grades. This situation impedes progress in expanding the range of steel grades, increasing casting speeds, improving slab quality, and further elevating the level of continuous casting [4,5,6,7]. Thus, designing high-performance mold fluxes for casting exceptional steel grades remains a challenge.

As exceptional steel grades continue to evolve, the development focus for mold fluxes has shifted towards product serialization, performance stability, and high compatibility with continuous casting process conditions. Kölbl et al. [8] developed a new mold flux formulation for the continuous casting of soft steel by optimizing the flux’s chemical composition and physical properties to improve the surface quality of the cast billets. Jiang et al. [9] investigated the reaction mechanism between high-Al steel- and CaO-SiO_2_-based mold flux and accordingly optimized the design of CaO-Al_2_O_3_-based mold flux to enhance the stability of the continuous casting process. Shen et al. [10] proposed an integrated research approach on the development and application of mold flux for high-speed continuous casting of high-carbon steel, achieving high production efficiency. Pereira et al. [11] designed and evaluated fluoride-free mold fluxes for peritectic steel continuous casting, aiming to reduce environmental hazards and optimize casting performance by replacing fluoride-containing fluxes. Therefore, addressing the application scenarios of mold fluxes in the continuous casting process of exceptional steel grades requires comprehensive technical analysis to identify the precise matching relationship between industrial steel grades and mold fluxes, to clarify the discrepancies between required and supplied flux properties, and to provide a basis for developing new mold fluxes suitable for continuous casting production requirements.

Hebei Iron and Steel Co., Ltd. (Tangshan, China) produces the low-alloy peritectic steel using the slab continuous caster. This steel grade possesses excellent impact toughness, wear resistance, and weldability, making it a type of high-strength structural steel widely used in manufacturing buildings, bridges, ships, pipelines, and automotive components [12,13,14,15]. However, phenomena such as surface cracks, slag entrapment, and sticker breakouts frequently occur during the continuous casting of this steel grade, to some extent reducing the comprehensive yield of slabs and increasing steelmaking production costs [16,17]. Tracing the root cause reveals that the issue is closely related to the mold fluxes used in continuous casting process. Many problems remain to be solved regarding the matching between mold fluxes and steel grades, as well as casting process conditions. Therefore, this study specifically targets the recurring issues in the continuous casting of low-alloy peritectic steel, which adversely affect slab quality and production efficiency. By systematically designing and evaluating mold fluxes with tailored physical properties and mineralogical characteristics, the aim is to develop high-performance mold fluxes that enhance both lubrication and heat transfer control during casting. This process directly supports the industrial application of low-alloy peritectic steels.

## 2. Experimental Section

### 2.1. Materials

**Figure 1:**
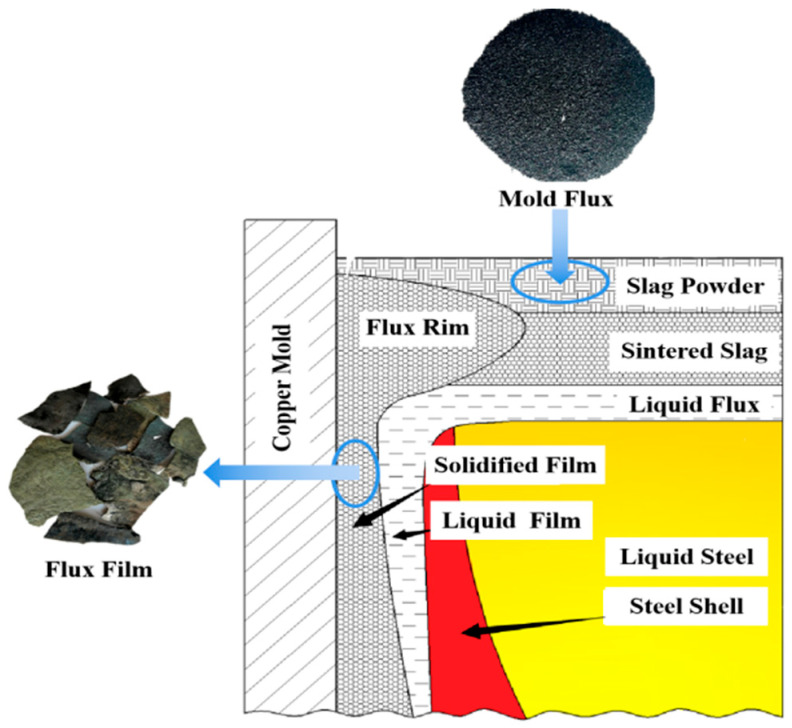
Position diagram of mold flux and flux film in the mold.

In this work, the mold flux and flux film samples required for the low-alloy peritectic steel continuous casting were provided by Hebei Iron and Steel Co., Ltd., Shijiazhuang, China. On-site tracking and investigation were conducted at the industrial production scenario to thoroughly examine the performance of low-alloy peritectic steel during continuous casting. Two typical and representative low-alloy peritectic steel grades (denoted as Steel A and Steel B) were selected, and corresponding mold flux samples (denoted as Flux A and Flux B) as well as their solidified flux film samples were successfully obtained. Moreover, the flux film samples were retrieved from the specific region adjacent to the mold wall during a routine interruption of the continuous casting process, ensuring that the acquired samples accurately reflect the actual state of the flux film during operation. The distribution of mold flux and the sampling position of the flux film in the mold are illustrated in Figure 1. The chemical compositions of the mold flux and flux film samples for low-alloy peritectic steel continuous casting are presented in Table 1.

### 2.2. Methods for Testing Physical Properties of Mold Flux

The melting point tester (KFMP-1600A, Kefeng Metallurgical New Materials Co., Ltd., Luoyang, China) was used to observe the high-temperature melting process of the mold flux samples in situ, following the Chinese Industrial Standard (GB/T 40404-2021) [18]. During the experiment, the sample was heated at a rate of 10 °C/min. The temperatures at which the sample height reduced to 3/4, 2/4, and 1/4 of its original height were recorded and defined as the softening temperature, hemispherical temperature (melting point), and flow temperature of the sample, respectively.

Following the Chinese Industrial Standard (YB/T185-2017) [19], the high-temperature viscometer (RTW-13, Northeastern University, Shenyang, China) was used to measure the viscosity data of the test fluxes during cooling, construct viscosity–temperature curves, analyze the break temperature where viscosity begins to change abruptly, and record the viscosity value at 1300 °C to characterize the viscosity properties of the test fluxes.

The high-temperature in situ thermal analyzer (S/DHTT-TA-III, Chongqing University, Chongqing, China) was used to observe the crystallization process of mold flux samples under different cooling rates and target temperatures. By identifying significant changes in slag surface morphology and light transmittance, the temperature–time points for the start and end of crystallization were accurately determined. These data allowed the construction of continuous cooling transformation (CCT) curves and isothermal transformation (TTT) curves for the test fluxes. Key crystallization parameters, such as the critical crystallization cooling rate and initial crystallization temperature, were analyzed from these curves. The selection of these parameters was based on reproducible inflection points in the curves, which reflect the initiation and persistence of crystallization, rather than arbitrary temperature increments. This approach ensures accuracy and consistency in characterizing crystallization behavior.

### 2.3. Methods for Analyzing Mineralogical Characteristics and Mechanism of Flux Film

Part of the flux film samples from each group were bonded together using epoxy adhesive. Sections along the thickness direction were mounted on glass slides, ground to a thickness of 0.03 mm, and polished to prepare thin sections for microscopy. Other flux film samples were completely ground to a powder state with a particle size of 0.074 mm. Polarizing microscope (Axio Scope A1 pol, Carl Zeiss AG, Oberkochen, Germany) was used to observe the thin sections of the flux film samples to analyze mineralogical characteristics such as mineral composition, crystallization ratio, and microstructure. The crystalline phase composition of powdered flux films was detected using an X-ray diffractometer (D8 Advance, Bruker AXS, Bremen, Germany) within a scanning range of 10° to 90°.

FactSage 8.0 software was used to simulate the equilibrium crystallization process of the flux films. Key parameters such as the types of precipitated minerals, precipitation temperatures, and precipitation amounts during crystallization were obtained by plotting multi-component multi-phase equilibrium diagrams using the Equilib calculation module. This study explored the crystallization behavior of each mineral phase and provided an in-depth analysis to elucidate the formation mechanisms of the flux film mineralogy.

Following the Chinese Industrial Standard (YB/T4130-2018) [20], the flat-plate thermal conductivity meter (PBD-13-4P, LIRR, Luoyang, China) was used to measure the changes in the thermal conductivity of solid flux film samples within the temperature range of 200 °C to 600 °C. Combined with the mineralogical structure characteristics of the flux films, this helped analyze the heat transfer mechanism of the flux film mineralogy.

## 3. Results and Discussion

### 3.1. Melting Properties of Mold Flux

**Figure 2:**
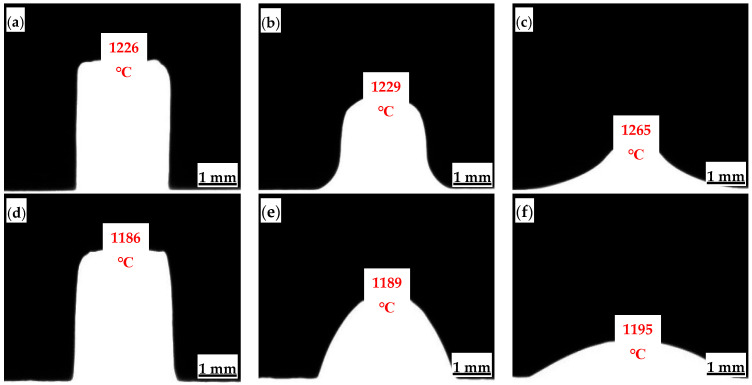
Melting temperatures of mold flux for low-alloy peritectic steel: (**a**) Flux A-softening point; (**b**) Flux A-hemispherical point; (**c**) Flux A-flow point; (**d**) Flux B-softening point; (**e**) Flux B-hemispherical point; (**f**) flux B-softening point.

Melting temperature and viscosity are two fundamental indicators of mold flux melting properties, significantly impacting the stability of the continuous casting process and the slab surface quality [21,22,23,24]. The melting point tester was used to observe the high-temperature melting process of the mold flux samples and record the softening temperature, hemispherical temperature, and flow temperature (Figure 2). The two mold fluxes studied for low-alloy peritectic steel casting have different melting temperature ranges. The melting temperature range for Flux A is 1226–1265 °C, while for Flux B it is 1186–1195 °C. It can be seen that all melting temperature indicators for Flux B are lower than those for Flux A, and its melting temperature range is narrower.

**Figure 3:**
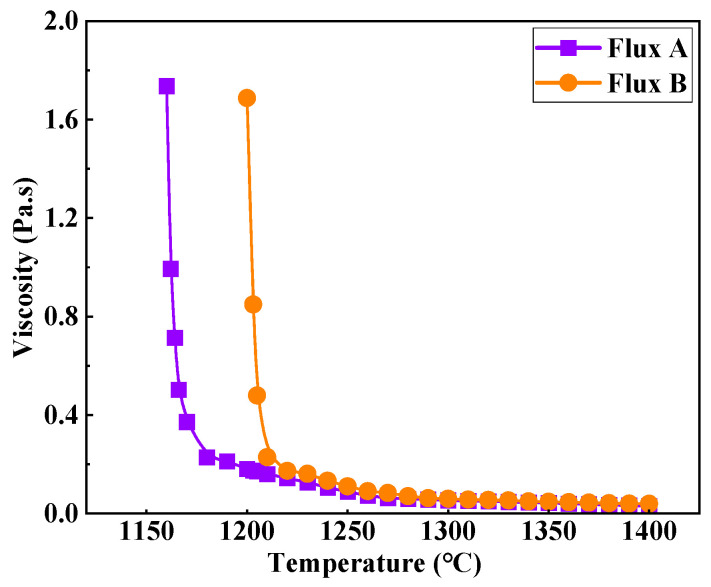
Viscosity properties of mold flux for low-alloy peritectic steel.

Figure 3 shows the viscosity–temperature curves of the two industrial mold fluxes for low-alloy peritectic steel. As the temperature decreases gradually from 1400 °C, the viscosity of both industrial fluxes initially increases slowly. Upon reaching a specific temperature (break temperature), the viscosity value begins to rise sharply. Flux A has a viscosity of 0.055 Pa·s at 1300 °C and a break temperature of 1180 °C. Flux B has a viscosity of 0.060 Pa·s at 1300 °C and a break temperature of 1210 °C. Although the viscosity values of Flux A and Flux B at 1300 °C are similar, the break temperature of Flux B is 30 °C higher than that of Flux A. Based on silicate slag structure theory, this is likely due to the slightly higher binary basicity and main flux content (Na_2_O) of Flux B compared to Flux A. This compositional feature enhances the complexity of the silicate network structure, facilitating easier crystallization of the flux and leading to an earlier onset of the viscosity break point and a more abrupt increase in viscosity.

### 3.2. Crystallization Behavior of Mold Flux

**Figure 4:**
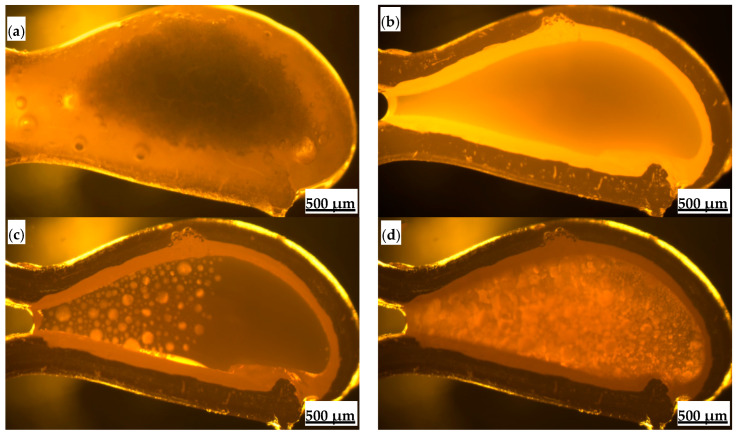
In situ observation of the melting and crystallization process of mold flux for low-alloy peritectic steel: (**a**) melting begins; (**b**) melting completes; (**c**) crystallization begins; (**d**) crystallization ends.

**Figure 5:**
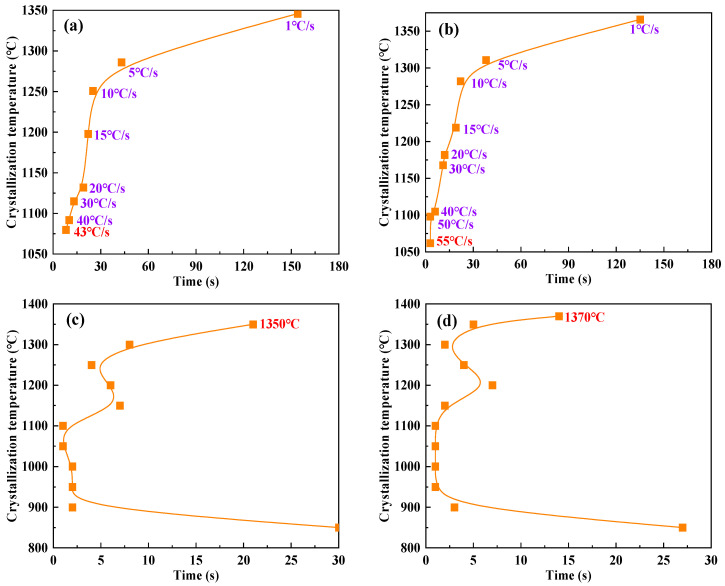
Crystallization behavior of mold flux for low-alloy peritectic steel: (**a**) Flux A-CCT curve; (**b**) Flux B-CCT curve; (**c**) Flux A-TTT curve; (**d**) Flux B-TTT curve. The orange squares represent data from crystallization experiments of continuous cooling transformation and isothermal transformation. The red texts highlight the critical crystallization cooling rate and the initial crystallization temperature achieved from CCT curves and TTT curves.

Crystallization behavior indicators, such as critical crystallization cooling rate, initial crystallization temperature, and crystallization incubation time, control the formation characteristics and structure of the flux film between the mold and strand. They are crucial parameters for coordinating heat transfer and lubrication [25,26,27,28]. The high-temperature in situ thermal analyzer was used to observe the entire process of melting and crystallization of the mold flux samples (Figure 4). Under experimental conditions of continuous cooling transformation and isothermal transformation, the continuous cooling transformation (CCT) curves and isothermal transformation (TTT) curves of the mold fluxes were constructed (Figure 5), ultimately allowing the accurate determination of crystallization behavior parameters such as the critical crystallization cooling rate and initial crystallization temperature.

The CCT curves show that as the cooling rate gradually increases, the crystallization temperatures of both Flux A and Flux B exhibit a clear decreasing trend. Under the same cooling rate, the crystallization temperature of Flux B is slightly higher than that of Flux A, and the critical crystallization cooling rate of Flux B is significantly greater than that of Flux A. The TTT curves show that no high differences can be observed in the crystallization temperature ranges and initial crystallization temperatures of Flux A and Flux B. However, under identical isothermal conditions, the crystallization incubation times of Flux B are generally shorter than those of Flux A. The combined experimental results indicate that Flux B, compared to Flux A, maintains a higher crystallization temperature even at higher cooling rates, demonstrating stronger crystallization capability. Furthermore, the shorter crystallization incubation time of Flux B means its crystallization process is faster, which is beneficial for rapidly forming a stable flux film during continuous casting, thereby optimizing heat transfer and lubrication effects.

### 3.3. Mineralogical Structure of Flux Film

The flux film formed when mold flux flows into the gap between the mold wall and the solidifying shell. The mineralogical structure characteristics generated during the solidification of this flux film are also key factors determining heat transfer uniformity and slab crack incidence [29,30,31,32,33]. This work employed polarizing microscopy combined with X-ray diffraction (XRD) to identify and analyze the microstructural characteristics of industrial flux films, focusing on the composition, content, morphology, and size of crystalline minerals.

**Figure 6:**
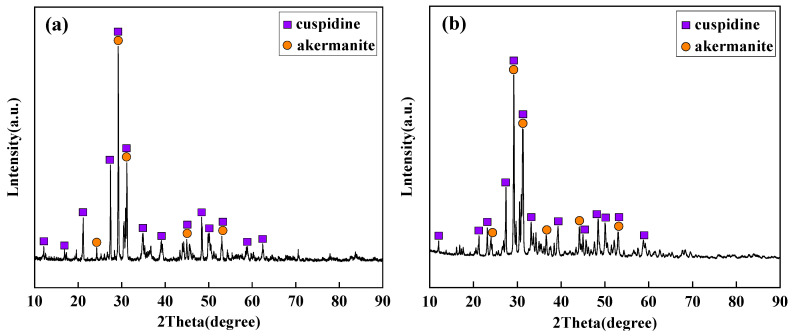
XRD analysis results of flux film for low-alloy peritectic steel: (**a**) Flux A; (**b**) Flux B.

**Figure 7:**
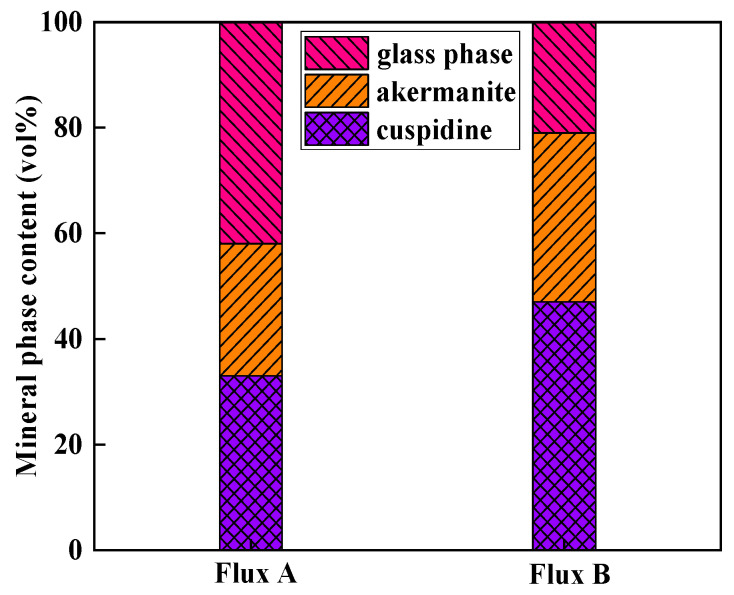
Mineral phase content of flux film for low-alloy peritectic steel.

Based on the identification results from polarizing microscopy and XRD (Figure 6 and Figure 7), the main crystalline phases in the industrial flux films corresponding to Flux A and Flux B are cuspidine and akermanite, with crystallization ratios concentrated between 60% and 80%. Compared to Flux A, Flux B’s flux film exhibits a stronger ability to precipitate cuspidine and akermanite, especially with cuspidine content approximately 15% higher, resulting in an overall higher crystallization ratio for the flux film.

**Figure 8:**
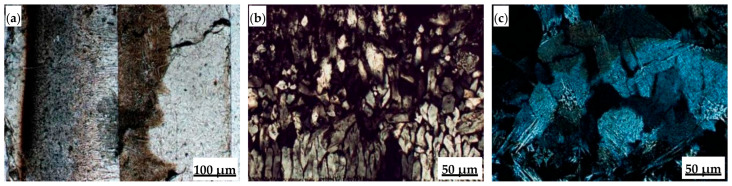

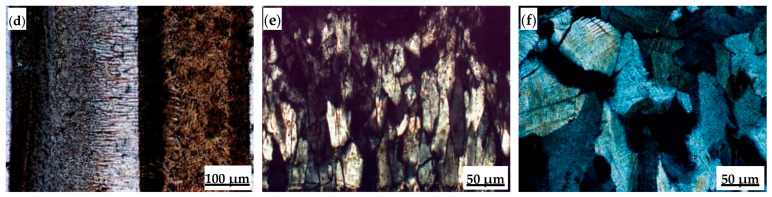
Microstructure of flux film for low-alloy peritectic steel: (**a**) film structure of Flux A; (**b**) spearhead-shaped cuspidine of Flux A; (**c**) woven-textured akermanite of Flux A; (**d**) film structure of Flux B; (**e**) platy cuspidine of Flux B; (**f**) woven-textured akermanite of Flux B.

Polarizing microscopy was used to analyze and determine microstructural characteristics such as layered structure, mineral morphology, and grain size of the flux films (Figure 8). The flux film of Flux A presents a typical “glass layer-crystalline layer-glass layer” three-layer structure. A distinct boundary within the middle crystalline layer divides it into a cuspidine layer and an akermanite layer. Cuspidine mostly appears as spearhead-shaped aggregates, while akermanite mostly appears as finely woven aggregates. The flux film of Flux B presents a typical “glass layer-crystalline layer” two-layer structure. The crystalline layer is further divided into a cuspidine layer and an akermanite layer. Cuspidine precipitates in large quantities, primarily as well-crystallized coarse spearhead-shaped and platy crystals; akermanite appears mainly as granular and woven aggregates. The morphological characteristics of crystalline phases are crucial for regulating the flux film’s heat transfer properties. Indeed, the coarsened and well-developed crystals, especially the interlocking platy-shaped and woven-textured crystals in Flux B, create massive micropores and complex grain boundaries in the crystalline layer, increasing interfacial thermal resistance.

The research results indicate that although the solidified flux films of different composition fluxes exhibit differences in mineral morphology and grain size characteristics, the layered distribution pattern of the mineralogical structure within the flux film is consistent. When liquid slag flows into the gap between the mold and strand, the side near the mold wall rapidly solidifies due to chilling, forming the glass layer of the flux film first. The contact between the glass layer and the mold wall creates significant interfacial thermal resistance, leading to a devitrification phenomenon in the glass layer. The crystals initially precipitating within this glass layer are primarily cuspidine. As the temperature drops rapidly on the strand side, the liquid flux film begins to solidify, precipitating akermanite crystals or forming another new glass layer.

### 3.4. Mineralogical Formation Pattern of Flux Film

**Figure 9:**
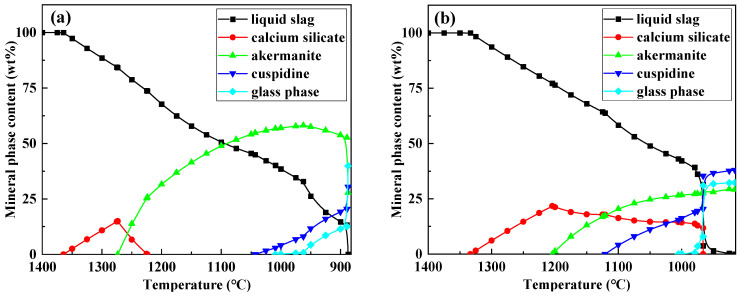

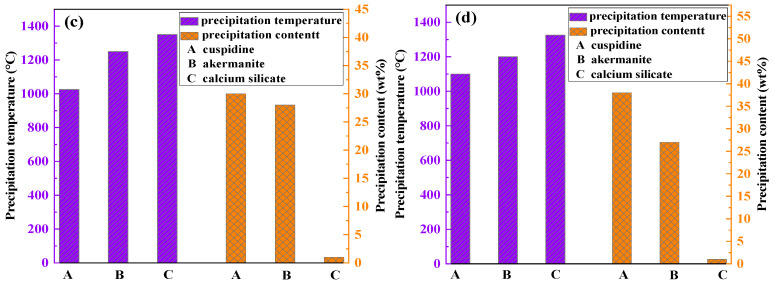
Crystallization process simulation of flux film for low-alloy peritectic steel: (**a**) Flux A—multi-component multi-phase equilibrium diagram; (**b**) Flux B—multi-component multi-phase equilibrium diagram; (**c**) Flux A—key parameters of precipitated minerals; (**d**) Flux B—key parameters of precipitated minerals.

To further elucidate the formation mechanism of flux film mineralogy, based on the chemical composition characteristics of the industrial mold fluxes, FactSage software was used to simulate the equilibrium crystallization process for Flux A and Flux B. Multi-component multi-phase equilibrium diagrams were plotted using the Equilib calculation module, and key parameters such as the types of precipitated minerals, precipitation temperatures, and precipitation contents during crystallization were obtained (Figure 9).

The crystallization process simulation results show that as the temperature decreases, the amount of liquid slag for both Flux A and Flux B begins to decrease around 1350 °C. At this stage, the intermediate phase calcium silicate precipitates. When the temperature drops below 1300 °C, the crystalline mineral akermanite begins to precipitate. When the temperature drops below 1150 °C, the crystalline mineral cuspidine also begins to precipitate. When the temperature continues to drop to 1000 °C, the glass phase begins to precipitate in large quantities, after which the liquid slag amount decreases sharply until it completely transforms into solid phases. The precipitation sequence of mineral phases during crystallization is consistent for Flux A and Flux B. However, differences exist in the precipitation temperatures and amounts of each mineral phase, indicating that the composition of the mold flux significantly influences the formation behavior of flux film mineralogy. Therefore, by adjusting the composition of the mold flux, the formation of an ideal mineralogical structure in the flux film can be optimized.

### 3.5. Mineralogical Heat Transfer Mechanism of Flux Film

**Figure 10:**
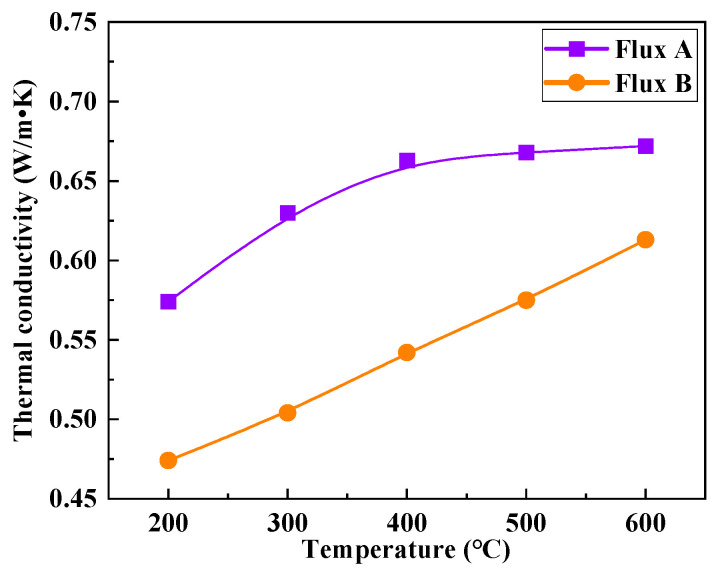
Relationship between temperature and thermal conductivity.

The thermal conductivity of the mold flux film is an important parameter characterizing its heat transfer performance [34,35]. Since the temperature of the solid flux film near the mold wall side is within the range of 200–600 °C, studying thermal conductivity under this temperature gradient is key to understanding the heat transfer mechanism. Standard samples of solid flux films for Flux A and Flux B were prepared using a mold flux film simulation device. The thermal conductivity meter was used to measure the variation in thermal conductivity of the flux film samples within the 200–600 °C temperature range (Figure 10). The experimental results show that as the temperature rises from 200 °C to 600 °C, the thermal conductivity of the flux films for both Flux A and Flux B gradually increases, roughly within the range of 0.47–0.67 W/m·K. At the same temperature, the thermal conductivity of Flux B’s flux film is significantly lower than that of Flux A, indicating that Flux B has a better ability to reduce the heat transfer rate within the mold. Furthermore, the change in thermal conductivity of Flux B’s flux film with increasing temperature is more linear, suggesting it has advantages in controlling heat transfer uniformity and stability.

**Figure 11:**
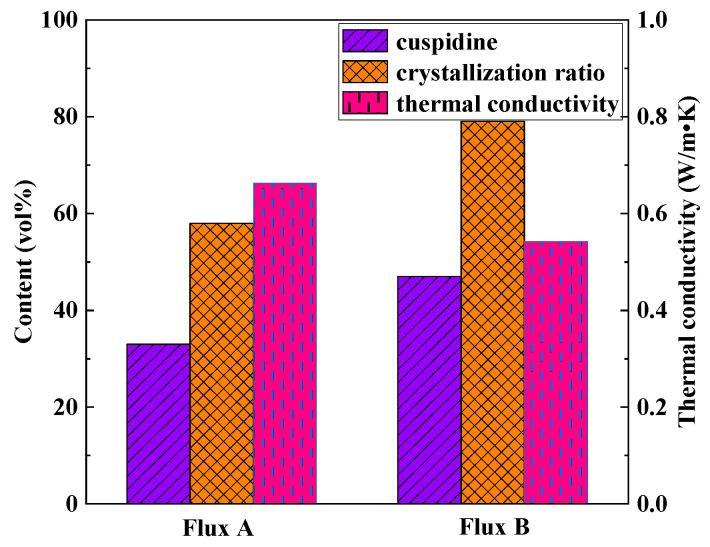
Relationship between mineralogical structure and thermal conductivity.

Figure 11 illustrates the relationship between the mineralogical structure and thermal conductivity of the flux films for the two industrial mold fluxes. Compared to Flux A, Flux B’s flux film mineralogy features a higher cuspidine content. High cuspidine content in the flux film usually also implies a higher crystallization ratio, but the corresponding flux film thermal conductivity is lower. Analysis suggests two reasons: Firstly, among the main crystalline minerals in mold flux, cuspidine has a lower thermal conductivity than other phases like akermanite, effectively reducing the overall thermal conductivity of the flux film. Secondly, the abundant precipitation of cuspidine in the flux film can easily trigger excessive crystal growth and coarsening of morphology, potentially leading to an increase in interfaces between crystals and the formation of more micropores around them, thereby significantly reducing the thermal conductivity of the flux film.

### 3.6. Discussion

**Figure 12:**
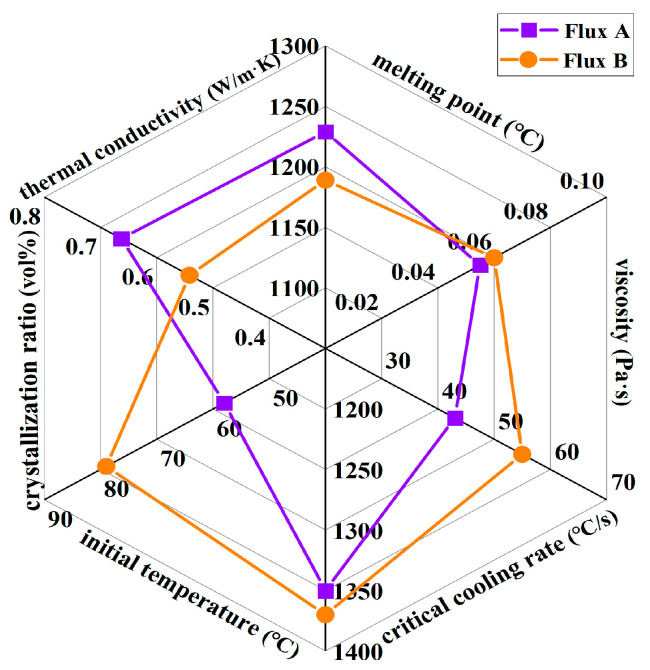
Key performance parameters of two mold fluxes for low-alloy peritectic steel.

Through systematic research on the physical properties and mineralogical characteristics of the industrial mold fluxes (Flux A and Flux B) for low-alloy peritectic steel continuous casting, the differences between the two mold fluxes in terms of melting point, viscosity, critical crystallization cooling rate, crystallization temperature, crystallization ratio, and thermal conductivity can be clearly defined (Figure 12). Their impact mechanisms on slab quality can be correlated.

The melting point of Flux B is significantly lower than that of Flux A, and its melting temperature range is narrower. This melting characteristic allows Flux B to form a more uniform liquid flux layer more easily within the mold, benefiting strand shell lubrication and reducing the risk of slag entrapment. Moreover, the low melting point (<1200 °C) of mold fluxes aligns with the recommended melting properties for peritectic steel casting highlighted by Mills [1] and Long [2].

Although the viscosity values of Flux A and Flux B at 1300 °C are similar, the break temperature of Flux B is 30 °C higher than that of Flux A. Drawing upon slag structure theory, the elevated binary basicity and Na_2_O content in Flux B enhance the complexity of the silicate network structure, leading to a significant increase in viscosity at higher temperatures. This viscosity characteristic may affect the fluidity of the slag in the high-temperature zone, increasing the risk of lubrication deterioration for the strand.

Flux B has a higher critical crystallization cooling rate (>50 °C/s), and under the same cooling rate, its crystallization temperature is higher than that of Flux A. This indicates that Flux B can still crystallize rapidly under high casting speed conditions, forming a stable crystalline layer in the flux film, which aids in uniform heat transfer. The crystallization incubation time of Flux B is shorter than that of Flux A, meaning its crystallization rate is faster, enabling rapid formation of the flux film structure and reducing instability of the liquid flux film, thereby lowering the incidence of sticker breakouts.

The crystallization ratio of Flux B’s flux film is significantly higher than that of Flux A, with cuspidine content increased by approximately 15%. This mineralogical structural feature leads to the formation of more micropores and grain boundaries, thereby increasing the overall thermal resistance of the flux film. Consequently, the thermal conductivity of Flux B’s flux film is markedly lower than that of Flux A, which can slow down the cooling rate of the strand shell in the mold, alleviating the volumetric shrinkage stress during the solidification of low-alloy peritectic steel, and thus inhibiting the formation of surface cracks.

The high critical crystallization cooling rate (>50 °C/s) and prevalence of cuspidine are consistent with the findings of Zhang [27] and support the theory that high basicity and Na_2_O content enhance crystallization tendency. The identified low thermal conductivity range (0.47–0.67 W/m·K) and its correlation with a high crystallization ratio and coarse crystal morphology, which increase thermal resistance by introducing micropores and interfaces, are in strong agreement with the mechanisms described by Mills [3] and Zhou [21].

In summary, Flux B outperforms Flux A in terms of melting properties, crystallization behavior, and heat transfer performance, making it more suitable for the efficient continuous casting of low-alloy peritectic steel. The higher Na_2_O content and basicity of Flux B are key to its optimized performance. In this work, through multi-faceted linkage analysis, the mapping relationship between mold flux compositions, physical properties, mineralogical characteristics, and slab quality has been clarified, providing a basis for the precise design of mold fluxes for low-alloy peritectic steel continuous casting. Therefore, the economic and sustainable approach lies in identifying the precise matching relationship between industrial steel grades and mold fluxes, which is achieved by optimizing mold flux compositions to enhance lubrication and heat transfer performance, thereby reducing slab defects. Future mold flux development needs to further balance the contradiction between lubrication and heat transfer to adapt to higher casting speeds and more demanding steel grade requirements.

## 4. Conclusions

This study was conducted with the explicit aim of optimizing mold flux design for low-alloy peritectic steel continuous casting by systematically investigating the physical properties of industrial mold fluxes and the mineralogical characteristics of their flux films. The following conclusions were drawn:

(1) Mold fluxes designed for low-alloy peritectic steel should possess a narrow melting temperature range, low melting point (<1200 °C), and low viscosity (<0.1 Pa·s) to facilitate the formation of a uniformly flowing liquid slag layer and enhance lubrication effectiveness.
(2) Strong crystallization capability of mold fluxes for low-alloy peritectic steel is essential, characterized by a high critical crystallization cooling rate (>50 °C/s) and a high initial crystallization temperature (>1350 °C). These properties ensure the rapid formation of a stable flux film structure and enhance heat transfer uniformity.
(3) The mineralogical structure of the flux film for low-alloy peritectic steel presents a multilayered structure with a high crystallization ratio (60–80 vol%), mainly composed of the crystalline minerals cuspidine and akermanite. The high abundance and coarsened morphology of cuspidine crystals play a key role in regulating heat transfer.
(4) The thermal conductivity of flux film for low-alloy peritectic steel remains low (0.47–0.67 W/m·K), primarily due to the high crystallinity and coarse crystal morphology that promote the formation of numerous micropores and grain boundaries between crystals, increasing the overall thermal resistance of the flux film.

## Figures and Tables

**Table 1:** Chemical compositions of mold flux and flux film for low-alloy peritectic steel (wt%).

Steel Number	Flux Number	CaO	SiO_2_	Al_2_O_3_	MgO	Fe_2_O_3_	K_2_O + Na_2_O	MnO	F^−^	C
Steel A	Flux A-mold flux	36.29	31.77	4.18	3.72	1.03	7.82	1.80	6.87	6.31
Steel A	Flux A-flux film	40.39	32.46	5.29	4.21	0.55	10.29	1.43	5.96	---
Steel B	Flux B-mold flux	38.31	31.03	4.32	1.55	2.03	8.78	0.32	6.80	7.03
Steel B	Flux B-flux film	44.69	31.55	5.30	2.00	0.49	9.51	0.17	5.76	---

## Data Availability

The data presented in this study are available on request from the corresponding author due to them being part of an ongoing study.
